# Current therapies in alleviating liver disorders and cancers with a special focus on the potential of vitamin D

**DOI:** 10.1186/s12986-018-0251-5

**Published:** 2018-02-09

**Authors:** Shahida Khan, Ashraf Ali, Sarah Khan, Ahmed Bakillah, Ghazi Damanhouri, Aziz Khan, Ahmed Makki, Ibtehal AlAnsari, Naheed Banu

**Affiliations:** 1 Applied Nutrition and Natural Products Group, King Fahd Medical Research Center, King Abdulaziz University, P.O.Box 80216, Jeddah, 21589 Kingdom of Saudi Arabia; 2 National Brain Research Center, Manesar, Gurgaon, 122051 India; 3 Department of Medicine, SUNY Downstate Medical Center, 450 Clarkson Avenue, Brooklyn, NY 11203 USA; 4 Department of Biochemistry, College of Medical Rehabilitation, Buraydah, Qassim University, P.O. Box 51452, Qassim, Kingdom of Saudi Arabia

**Keywords:** Vitamin D, Liver disorders, Anti-inflammatory, Current therapies

## Abstract

**Background:**

Liver dysfunction is a topic of global concern with many advancing therapies being researched. Though vitamin D takes a center place, other therapies especially nutritional are also gaining ground. Vitamin D has gone beyond its role in skeletal disorders by showcasing its associations in other metabolic dysfunctions too.

**Result:**

Epidemiological evidences show a correlation between the status of vitamin D and different forms of cancer. Vitamin D receptors and alterations in gene expression appear decisive in the development of chronic liver disorders. Nutritional status therefore plays a significant role in avoiding the complications related to liver dysfunctions, making it mandatory in maintaining vitamin D sufficiency in the body. Therapies with omega-3 fatty acids, antioxidants, amino acids, steroids also render benefits which could be further explored. Recent research on the progression of certain forms of liver cancer using vitamin D analogs like Seocalcitol EB 1089 has shown good promise.

**Conclusion:**

The anti-inflammatory and immuno- regulatory properties of vitamin D makes its analogs, suitable candidates of better choice for the prevention and treatment of liver disorders and cancer.

## Background

Vitamin D is an ecosystem dependent vitamin and any degradation by way of environmental pollution affects the availability of this vital nutrient. Vitamin D has been foreshown as a key component linking it to various medical problems. The presence of the precursor cholecalciferol can be seen in non-mineralized 750 million-year-old plankton. The dynamism of this molecule and its receptor has roots in the evolutionary and chemical processes of life with the specific receptor predicting absolute effects in biologically complex ways [1].

With vitamin D deficiency rapidly becoming widespread in Asia [2], Gulf regions [3, 4] and in European countries, its value is being re-recognized. It has emerged as a novel player in benefits to the heart, insulin secretion control, anti-microbial defense, detoxification of xenobiotics, and, possibly, anti-proliferative, and apoptosis exhibiting immuno-modulatory functions in different cancers. Lower risks to some cancers in China have been linked to UVB radiation responsible for vitamin D production [5]. Vitamin D has now gone beyond the role of humeral homeostasis and osseo beneficial effects. The relationship between UVB exposure and vitamin D with cancer has many supportive evidences from observational [6] ecological [7] and randomized controlled trials [8].

An association between concentrations of vitamin D and occurrence as well as severity of chronic liver disease (CLD) has been well established as seen in Table 1. Genetic disposition to metabolize vitamin D appears to be decisive in the severity of CLD [9] (Table 1). Other associations of vitamin D have been attributed to the development and evolution of non-alcoholic fatty liver disease (NAFLD) and chronic hepatitis C (CHC) virus infection [10].

**Table 1:** Some of the genes and their functions/organs regulated by vitamin D

Gene	Target Organ	Regulation	Function of genes involved
PTH	Parathyroid gland	down	Induces Cyp27B1 and inhibit 24-hydroxylation, increase calcitriol synthesis
Cyp24A1 (VD-24-hydroxylase)	kidney	up	Inactivates calcitriol to 1,24,25(OH)_3_D_3_, which can be oxidized to calcitriol acid for secretion
Interleukin −12(IL-12)	Innate Immune Cell	down	Restrain Th1 activation by limiting IL-12
FGF15 (FGF19 for humans)	Ileum	up	Suppresses bile acid synthesis in hepatocytes through induction of SHP
Cyp27B1 (VD-1alpha-hydroxylase	kidney	down	Restrains VD synthesis by feedback inhibition
Cyp3A4	liver		Catabolizes bile acid and xenobiotics,
SULT2A1	liver	up	Detoxification of steroids, xenobiotics, and bile acid for secretion
MRP3	liver	up	Transportations of range of substrates such as anti-cancer drugs and bile acid
Cathelicidin	intestine	up	immune defense against invasive bacterial infection
ASBT	Ileum	up	Enhances bile acid re-adsorption from ileum, which may restrain bile acid synthesis in the liver via feedback inhibition
Calbindin D9K	Intestine, Kidney	up	Transport of calcium across the enterocytes
TRPV6	intestine	up	Help in calcium absorption in the intestine
Defensin beta 2	Immune cells	up	Exhibits potent antimicrobial activity against Gram-negative bacteria and Candida, but not against Gram-positive *S. aureus*
FasL/CD95L	Immune cells	down	Reduces apoptosis
E-cadherin	skin	down	Restrains epithelial to mesenchymal transition
Interferon-gamma	T cells	up	Restrains immunity

Biochemical studies show the responsiveness of Hepatocellular carcinoma cells (HCC) to the various inhibitory effects by vitamin D as well its analogs [11]. Analogs of this vitamin have also been found to be potentially effective in alleviating problems related to pancreatic cancer [12] and cancers of the prostrate and liver [13]. The nuclear receptor of vitamin D (VDR) appears to play a significant role in homeostasis of the calcium signaling. However the mechanistic pathway of VDR on therapeutically liver functions needs to be elucidated [14]. Also there are some issues worth considering whether vitamin D deficiency is the cause or effect of the disease and if so how and why. Many studies have also showed that vitamin D supplementation is ineffective in ameliorating many diseases (Table 2) and it is not clear why this is so.

**Table 2:** Clinical and non-clinical effect of Vitamin D on various target organs

Function	Target Organ/Function	Reference
Inhibits in vitro HCV replication in a dose-dependent manner	Liver	[55]
Vitamin D supplementation may improve SVR rate in HCV among various genotypes	Liver	[128]
Low vitamin D level leads to non-responsiveness to interferon based treatment	Liver	[164–166]
Vitamin D deficiency causes downregulation of 25-hydroxylase CYP27A1 in, liver resulting in increased fibrosis and low SVR	Liver	[164, 167]
Deficiency causes increase in fibrosis and inflammation in liver	Liver	[164–166, 168, 169]
Supplementation/phototherapy improves liver histology in preclinical studies of NAFLD	Liver[NAFLD]	[170, 171]
Supplementation prevents liver fibrosis in preclinical studies	Liver[NAFLD]	[171, 172]
Low vitamin D level causes severe steatosis, inflammation and fibrosis	Liver[NAFLD]	[167, 169, 173, 174]
Vitamin D deficiency causes increased hepatic expression of TLR2, TLR4 and TLR9, which are implicated in NAFLD pathogenesis	Liver[NAFLD]	[174]
Vitamin D increases Ca and PO_4_ absorption from small intestine	Calcium and Bone	[175]
It Suppresses PTH secretion and Induces osteoclast maturation	Calcium and Bone	[175, 176]
Normal vitamin D level lowers prevalence of metabolic syndrome by 67%	Pancreatic functions	[177]
Supplementation improves insulin sensitivity and lowers risk of developing type 2 diabetes	Pancreatic functions	[178]
Vitamin D deficiency increases chances of insulin resistance	Pancreatic functions	[179]
Impaired pancreatic β-cell function in cases of low vitamin D level	Pancreatic functions	[180, 181]
Low level of vitamin D decreases macrophage TLR response and increase chances of TB infection	Innate Immunity	[182]
Vitamin D is essential for NK cell development and function	Innate Immunity	[183]
Vitamin D Inhibits proliferation of Th1 lymphocytes	Adaptive Immune System	[184]
Supplementation decreases risk of developing MS in women and type 1 diabetes in children	Adaptive Immune System	[177, 185]
Low vitamin D level increases risk of *M. tuberculosis* infection	Immunity	[186–189]
Normal Vitamin D level decreased population incidence of autoimmune diseases such as MS	Immunity	[190]
Higher 25(OH)D levels associated with lower incidence of colorectal adenoma	Carcinogenesis	[191]
Sunlight exposure associated with reduced risk of NHL	Carcinogenesis	[192]
Vitamin D decreased risk of colon, breast and prostate cancer	Carcinogenesis	[193–198]
Vitamin D deficiency is associated with bone weakness and painful crises	SCD	[199, 200]
vitamin D deficiency and its potential association with acute pain in SCD	SCD	[38]

*SVR* Sustained Virological Response, *NAFLD* Non-alcoholic fatty liver disease, *TLR* Toll like Receptor, *PTH* Parathyroid Hormone, *MS* Multiple Sclerosis, *NHL* Non-Hodgkin lymphoma, *SCD* Sickle Cell Disease

A recent randomized study of 409 elderly people in Finland suggested that vitamin D failed to offer any benefits compared to placebo or exercise-and that fracture rates were, in fact, slightly higher [15]. A few research findings peg the vitamin, as a consequence and not as a cause of poor health or a disorder, dispelling the notion that vitamin D deficiency could effect a string of such disorders. According to these researchers it’s essentially the disorder itself causing a deficiency of vitamin D [16]. A few researches contemplate that the increased inflammatory processes of the disorder itself may lead to the cause of the deficiency of vitamin D [17]. In one of the rarer RCT it is concluded that supplementation with vitamin D and Ca significantly reduces the risk of all kinds of cancers [18]. In another meta- analysis report, there was an inverse relationship between circulating vitamin D and risk of type 2 diabetes across different populations [19]. Vitamin D deficiency may not be helpful in many diseases and certain cancers but these negative studies could be helpful in redesigning appropriate trials in future by filling gaps of knowledge.

Emerging scientific research points towards vitamin D levels influencing CLD. In recent times vitamin D has been implicated in the complete cell cycle regulation [20]. Also a significant inhibition of lipid per oxidation thereby preventing cell damage due to free radical production, makes its usage prominent in HCC [21]. It has been observed that polymorphisms in the vitamin D binding protein (VDBP) are associated with alterations in vitamin D levels. A significant linkage has been found to exist between VDR polymorphism and incidence of HCC among liver cirrhosis patients. In a study conducted on a Chinese population, it was observed that polymorphism in VDR rs2228570a and DBP rs7041 are more responsible for higher susceptibility to hepatitis B virus related HCC [22]. Recent published trials showing the association between vitamin D and HCC have been documented in Table 3.VDR polymorphism is also associated with idiopathic short stature in several studies [23]. Recently, Marek et al. has conducted an important study linking growth hormone (IGF-1 axis), Vitamin D and bone mineral density (BMD) in patients afflicted with chronic viral hepatitis. They observed a positive correlation between bone mineral density and IGF-1, IGFBP-3 and BGLAP level. Chronic viral hepatitis leads to reduction of IGF-1 and IGFBP-3 and an increase in GH secretion [24] . In certain untreatable genotypes, pretreatment levels of vitamin D and VDBP predict sustained viral response after interferon and antiviral ribavirin therapy [25]. This was also observed earlier in other immune challenged patients [26] .

**Table 3:** Recent published trials on Vitamin D supplementation and HCC

Trial	Results	Reference
Association of Serum 25-Hydroxyvitamin D concentrations with certain circulating blood parameters in Cirrhotic Patients.	Though supplementation of vitamin D considerably enhances serum levels of 25(OH) D, no significant effect either on liver function tests and fibrosis or on PTH and mineral metabolites was observed.	[201]
Gene polymorphisms of the vitamin d receptor may prove a risk factor for development of HCC.	In chronic hepatitis C patients, the role of VDR ApoI polymorphism is crucial in the development of HCC	[202]
25-hydroxyvitamin D deficiency as a prognostic marker in patients with HCC.	25(OH) D3 is a marker metabolite for poor consequences and its deficiency is associated with advanced progression of HCC.	[203]
Association between circulating vitamin D levels and the risk of developing HCC amongst Europeans.	There is an inverse association between the 25(OH) D serum concentrations and the risk of HCC.	[204]
Mechanistic role of 1, 25(OH) 2D3 in the progression of hepatocellular carcinoma.	Decreased expression of HDAC2 and increased expression of p21 (WAF1/Cip1), repressed the progression of HCC on administration of 1, 25(OH) 2D3 suggesting a therapeutic role of the vitamin in potential drug developments.	[205]
Tapping the 25(OH) D-induced cell growth inhibition potential for future targeted therapy in HCC patients.	25(OH)D and CYP27B1 gene transfection therapy could well be targeted for future HCC management studies	[206]
Effect of SNPs in VDR and DBP genes on HBV linked HCC risk amongst Chinese.	Polymorphisms of VDR rs2228570 and DBP rs7041 may be responsible for an increased susceptibility to HBV-related HCC amongst Chinese residents.	[22]
Association of vitamin D and cytokine production in HCC	Up-regulation of p27(kip1) expression in immune cells and reduction in the production of cytokines may contribute towards the inhibition of HCC progression on administration of vitamin D	[207]
Use of vitamin D, IL-6, and IL-17 concentrations as potential biomarkers in HCC patients for a more favorable prognosis	Vitamin D and the cytokines IL-6and IL-7 could be additionally used as prognostic markers in HCV and cirrhotic patients to suppress the development of HCC in them.	[208]
Effect of vitamin D supplementation on levels of CYP24A1 mRNA in hepatocellular carcinoma cell lines.	In-vitro supplementation of 1, 25(OH) (2) D (3) results in marked increases of CYP24A1 mRNA expression in few, though not all, human HCC lines.	[209]

Hepatic osteodystrophy in children displays of vitamin D deficiency rickets, poor bone mineral density and fractures caused by nutritional imbalance and necessitates treatment with cholecalciferol (D3) or ergocalciferol (D2) [27]. This review discusses the various roles and pathway of vitamin D in CLD with a holistic preventive approach.

## Link of vitamin D and liver complications with other coexisting health disorders

Risk factors for CVD such as abdominal obesity, diabetes, hypertension and hyperlipidemia are frequently observed in the same person. Serum calcitriol was found to inversely correlate with blood pressure and concentrations of triglycerides and calcidiol with fasting levels of insulin and lipoprotein lipase activity in skeletal muscle and adipose tissue [27, 28]. Statins are known to exert beneficial effects, not only by lowering cholesterol but also control diabetes, bone metabolism, and, inflammation that are probably related to vitamin D metabolism dysregulation. Administration of atorvastatin was found to decrease cholesterol levels and elevate vitamin D levels, because of the commonality of the 7-dehydrocholesterol metabolic pathway [29]. Though new insights have pointed towards a therapeutic role of vitamin D in CLD, the exact mechanistic role has not been interpreted. Severe deficiency of this vitamin has resulted in poor prognosis in prospective hepatic carcinoma patients and infectious complications [30]. The results of these studies show that vitamin D supplementation may be beneficial for the management of liver disease at least in certain groups of patients.

Obese individuals with CVD and diabetic complications also exhibit vitamin D deficiency probably due to vitamin D sequestration in subcutaneous fat thereby making vitamin stores less available to be converted to their potent form. Also lesser outdoor activity, and vitamin D production impairment by the skin due to clothing habits may be the factors responsible for increased complications [31].

Vitamin D receptors, vitamin D binding protein, and some allelic gene variations have been shown to be linked to insulin sensitivity, insulin secretion, glucose intolerance and, inflammation [32]**.** Also an inverse association has been found between concentrations of HbA_1c_ in the elderly population and levels of 25(OH) D3 [33]. Vitamin D was thus found to influence many aspects of the metabolic syndrome, like hypertension, insulin resistance, hyperglycemia, and hyperlipidemia (see Table 2) [34].

Overexpression of VDR has been shown to increase dopamine (DA), neuroblastoma SH SY5Y cell neuron differentiation by increasing expression of tyrosine hydroxylase and also reducing expression of neurogenin 2, a marker of immature dopamine neuron [35] Human corneal epithelial cells (HCEC) also contain functional (active) vitamin D receptor. Microarray data showed that vitamin D regulates various genes in HCEC and affects the toll like receptor signaling via upregulation of the regulatory protein IκBα. [36] In addition L-thyroxine treatment has been shown to increase 25-hydroxyvitamin D and decrease PTH level in hypothyroid women with post-partum thyroiditis [37].

Several studies reported that vitamin D deficiency is associated with bone weakness and painful crises (see Table 2). The potential of vitamin D deficiency in health conditions from obesity to cancers has been well researched (see Table 2). Its implication in hematological disorders is also of great interest as the metabolic burden on liver may cause a decreased production of the binding protein of vitamin D. Vitamin D supplementation has also shown to improve vaso-occlusive crisis (VOC) and acute pain in sickle cell disease (SCD) [38, 39].

The regular functions of the Ca^2+^ signaling and ROS pathways are preserved by vitamin D, along with the nuclear factor-erythroid-2-related factor 2 -Nrf2, and klotho. A deficiency of this vitamin may lower the signaling, cause an increased generation of ROS and thereby account for the implication of this vitamin in so many disorders [40].

## Vitamin D deficiency in chronic liver diseases

Deficiency of vitamin D is commonly observed in CLD (see Table 2) and has been associated with the development and evolution of non-alcoholic fatty liver disease (NAFLD) and chronic hepatitis C (CHC) virus infection. The role of vitamin D in the pathogenesis of NAFLD and CHC is not completely known, but it seems that the involvement of vitamin D in the activation and regulation of both innate and adaptive immune systems and its anti-proliferative effect may explain its importance in these liver diseases. It has been seen that CLD patients are at very high risk of vitamin D deficiency regardless of their etiology or disease severity [10].

But, apart from its classical definition of calcium absorption, and deposition, the optimal level of vitamin D insufficiency or deficiency is not clear. A map developed by the International Osteoporosis Foundation classifies: serum level of < 25 nmol/L of 25-hydroxyvitamin D as deficient; 25–49 nmol/L as insufficient; and 50–74 nmol/L as inadequate. Optimal vitamin levels for healthy liver function, and threshold value for CLD are not yet defined [41]. Symptoms of the deficiency of vitamin D often appear so non-specific that attributing it to a particular disease becomes difficult. Moreover its increasing deficiency globally in recent times has led to scrutinize it with more research backed studies.

## Role of vitamin D in non-alcoholic steatohepatitis-

NAFLD is one of the most common liver disease globally, and characterised by excess lipid deposits that affects around 20–30% of adult population and 8% of adolescents. Insulin resistance along with other confounding causative factors such as obesity and type 2 diabetes appear to be implicated in this disorder. NAFLD progresses to NASH (non-alcoholic steatohepatitis) which is categorised by increased inflammation of the liver and excess fat deposits. Obese children displayed lower than 20 ng/ml of vitamin D concentrations indicating that body mass is significantly associated with deficiencies of this vitamin, though the exact pathway in NAFLD remains unclear [42]. Risk of NAFLD was 4.14 times more in overweight and 5.98 times more in obese adolescents, when compared with their counterparts with normal weight [43]**.**

A meta-analysis of approximately 5000 NAFLD cases and 8000 controls, showed that on an average, NAFLD patients exhibited 0.36 ng/mL lesser levels of 25(OH) D as compared to the controls and 26% of them were vitamin D deficient showing that this vitamin could be a part of the pathogenesis of NAFLD [44] . In NAFLD patients, 60% of triglyceride sources were shown to come from serum non-esterified fatty acids from diet, with only 26% triglyceride that were made via hepatic de novo synthesis indicating that high intakes of triglyceride is probably the major factor for the development of NAFLD [45]. High intake of emulsified fat by bile acid is probably the first step leading to excessive fat deposition in liver leading to NAFLD and NASH.

The farnesoid X receptor belongs to the nuclear receptor family and is expressed in organs like the kidney, liver, adipose tissue and intestine. It influences the synthesis of bile acids, cholic and chenodeoxycholic acids which is through two known pathways. The classical or neutral pathway instigated by 7α- hydroxylation produces both bile acids, whereas the acidic pathway commencing with the side chain hydroxylation (C27) produces only chenodeoxycholic acid. A major difference is that the key enzyme cytochrome P450 oxidase and the sterol 27-hydroxylase CYP27A1 of the acidic pathway are neither regulated by the bile acids nor its levels altered in FXR-deficient mice. But FXR is involved in the down-regulation of expression of the microsomal rate-limiting enzyme CYP7A1 of the neutral pathway promoting it as a potential therapeutic agent [46]. Hypovitaminosis D is associated with increased body fat mass, and greater severity of NAFLD [47]. Hypo vitaminosis in NAFLD patients’ needs to be demonstrated in future long term follow up studies to attribute a causal role to the vitamin, so that a therapeutic solution could be adopted.

## Vitamin D in alcoholic liver disease (ALD)

Alcoholic liver disease (ALD) is one of the major concerns of CLD, which can be responsible for fibrosis and cirrhosis if untreated. Report published by the National Institute of Health on Alcohol Abuse and Alcoholism, stated that liver cirrhosis is the 12th leading cause of death in USA, amounting to 48%. Vitamin D deficiency is also an issue in this disease, but its role need more clarification. Hepatic fibrosis, it has been found that the active form of vitamin D downregulates the expression of type 1 collagen (COLIα1 and COLIα2) in hepatic stellate cells in a time dependent manner [48]. It was also observed that among alcoholic cirrhosis patients, 80% had serum vitamin D levels below 50 nmol/L, and 55% of patients had vitamin D levels below 25 nmol/L [49]. Lack of vitamin D may cause abnormal and impaired catabolism of alcohol and excessive triglyceride accumulation in liver subjects. It is still debatable if alcohol impairs absorption or impedes 25- or 1-hydroxylation step for the synthesis of active vitamin D. A research study on pregnant Ukranian women with low basal concentrations of vitamin D showed that consumption of alcohol had deleterious effects on vitamin D status during low sun available seasons of winters and spring as compared to high sun available seasons of summer and fall [50]. In ALD it is hypothesized that vitamin D may modulate the early immune response by regulating the T cells namelyTh2 and Treg [51]. Another possibility might be attributed to the regulation of genes for alcoholic metabolism by vitamin D. Enhanced accumulation of triglyceride in the liver and irregular catabolism of alcohol, possibly resulting from a deficiency of vitamin D should therefore be duly addressed [52].

## Vitamin D in viral hepatitis

Though insufficiency and deficiency of vitamin D have been correlated with chronic hepatitis C (CHC), their innate mechanisms seem quite complex. The extra- and intracellular levels of hepatitis C virus (HCV) core antigen is decreased on supplementation with escalating doses of vitamin D, suggesting a natural antiviral role of the vitamin. Additionally a reduction of many pro-inflammatory factors like interleukin-17, interferon-γ and tumor necrosis factor-α was seen. The anti-fibrotic property of vitamin D could also be partially responsible for the benefits of this vitamin supplementation in CHC. Reduced vitamin D level is linked with severe fibrosis and lowered sustained virological response (SVR) rate in CHC patients [53]**.** Interferon therapy supplemented with vitamin D resulted in high SVR rate among CHC genotype 1 patients [54]. Surprisingly it is noted that it is not the 1, 25(OH) 2 D3 form, but the 25(OH) D3 form which was responsible for reduced levels of infectious amount of hepatitis C virus [55]. In addition, cholecalciferol has been shown to possess superior potentiation of the antiviral activity than α calcidiol only during the initial periods of combined Peg-IFN-α2a plus ribavirin therapy. This was through upregulation of serum 25(OH)-D3 levels [25]. Combined effects of circulating 25(OH) D and BAt (CCA) haplotype have been significantly linked with the advancement of liver fibrosis in patients with HCV in clinical experiments [56]. The majority of patients with HBV-infection and related disorders exhibit vitamin D deficiency and related adverse clinical effects, suggesting vitamin D supplementation as a supportive option [57].

## Possible route of vitamin D in liver cancer

Most of the patients with HCC display the presence of CLD’s of which 80% exhibit liver cirrhosis due to chronic infection of either hepatitis B or C virus, excessive alcohol intake, or other lifestyle and diet related disorders. As risk factors vary across globally geographic regions, a well-defined molecular classification would probably give us leads about the molecular aberrations, thereby associating specific mode of treatments. Also better and efficiently designed clinical trials could result in novel molecular targeted agents [58].

Liver appears to play a critical role in the hydroxylation process leading to the formation and efficient utilization of the active form of vitamin D [59]. Patients with CLD therefore exhibit hypocalcaemia and bone disease. Generally the concentration of 24- hydroxylase in serum is accepted as a standard marker in deciding vitamin D status in patients with a normal renal function. It has been observed that patients with CLD exhibiting alterations in vitamin D metabolic pathway also exhibit changes in the calcium homeostasis projecting clinical signs for hepatic osteodystrophy [60].

Two hydroxylation steps are needed to transform vitamin D absorbed through the skin or consumed through diet to the bio-active form1α,25-dihydroxyvitamin D3 (1,25di(OH)-D3) also known as calcitriol. The first hydroxylation takes place in the liver by the enzyme 25-hydroxylase. The hydroxylation finally to the active metabolite takes place in the kidney by two renal enzymes 25-hydroxyvitamin D 1α-hydroxylase, and 1, 25-dihydroxyvitamin D 24-hydroxylase**.**Serum endocrine 1, 25-D3 levels, and mineral homeostasis are controlled by these renal hydroxylases. Levels of 1, 25(OH) 2D3, in the target cells retaining the hormone are expressed through nuclear vitamin D receptor (VDR). Many other cell types like the pancreas skin, vasculature, colon and immune system cells expressing VDR may also produce extra-renal 1,25(OH)2D3 [14, 61]. It has been observed that TNF alpha enhances the responsiveness of keratinocytes toward production of calcitriol, by upregulating VDR receptor through increased m RNA levels [62].

It has also been noted that decreased serum levels of vitamin precursor 25- hydroxyvitamin D3 (25-D3), predisposes one to different chronic diseases, and cancers rather than the active form of the vitamin. In cancer patients, the autocrine/paracrine extra-renal vitamin D system has the ability to synthesize and locally degrade the active form of 1,25- D3 which is essential for maintaining normal cell growth and resisting the mitogenic stimulus [59].

In cancer cells, the active form of vitamin D is rendered inactive in the presence of the enzyme 24-hydroxylase. Patients experiencing CLD, carcinoma, cirrhosis, ALD, and bile stone formation exhibit low serum concentrations of 24- hydroxylase [60]. Presence of extra-renal 24- hydroxylase is observed in cancer cells and is being recognized as rate limiting in relation to vitamin D levels. Upregulation of this enzyme extra-renally may act as a marker of vitamin D requirement in cancer patients and might be used as an adjunct medicine [63]. Earlier study shows a correlation between the enzyme 24- hydroxylase, severity of hepatic dysfunctions and mortality in CLD patients [64]. Though many studies demonstrate the anti-proliferative as well as pro-apoptotic effects of vitamin D in different tumor model systems in vitro and in vivo, clinical trials supporting this view are wanting. Recently published trials have been enlisted in Table 3. Liver cytochrome P450 enzymes (CYP27A1 and CYP2R1) and the kidney cytochrome P450 enzymes (CYP27B1) are responsible for converting the precursor to the active form of vitamin D metabolite. The ligand dependent nuclear transcription receptors PPAR alpha, gamma, HNF-4alpha and SHP seem to regulate CYP27A1. Possibly an overexpression of the enzyme CYP24A1 in different tumors, rapidly leads to decreased bioavailability and inactivation of 1, 25 D3 locally. Preclinical studies revealed that CYP24A1 inhibitors can suppress the breakdown of 1,25D3 in tumor cells and enhance the anti -tumor effects of 1,25D3 on cell growth and gene expression. A combination of CYP24A1 inhibitors and 1,25D3 may cause hypercalcemia in some patients which was found to be significantly reduced on administering dexamethasone. Furthermore dexamethasone was also found to enhance the antitumor potential of 1,25D3, increase VDR-ligand binding, and enhance VDR protein expression**.** Strategies exploiting the combinational effects of CYP24A1 inhibitors and dexamethasone are in progress [65] **.**

In a cohort of G1CHC patients, lower hepatic VDR expression of VDR protein has been found to be associated with the severity of both liver fibrosis and inflammation [66]. Therefore randomized treatments with vitamin D3 supplementation have been proposed to assess the role of this vitamin [67].

One of the key molecules in the inhibition of hepatoma tumor progression is dermatopontin (DPT). It is mainly found in the cytoplasmic portion of different liver cells.The extra cellular region of the skin also contains DPT. DPT has been recently identified as a downstream target of VDR. Experiments using the DPT homolog MCTx-1 of Millepora origin has been found to be a potential cytotoxin in leukemia. Expression of DPT mRNA was high not only in human liver, but also in many organs like the spleen and kidney, and to a lesser extent in heart, and the ovary, and almost absent in all other tissues and HCC cells. The increased presence of TGF-beta1 in HCC tumor cells suggests one of its possible role in the development of HCC along with possibly other mechanisms [68] wherein DPT could be used.

## Pivotal role of vitamin D receptors (VDR) and gene expression

VDR is a protein coding gene also known as calcitriol receptor. It is a member of the nuclear receptor family of transcription factor, located on chromosome 12.VDR is a regulator of gene transcription that is found in the nucleus of cells. Its presence is noticeable largely in the bone, intestine and kidney and to lesser extent in many other tissues [69]. VDR has been known to be critical in not only maintaining bone metabolism through calcium and phosphate homeostasis, but also in immune-modulation, cell development and differentiation [70] .

VDR is present in the cytosol and gets translocated to the nucleus where it binds with a ligand resulting in a heterodimer along with another nuclear receptor, the retinoid X receptor RXR. This RXR influences the effects of retinoids through retinoic acid mediated gene activation. This heterodimer subsequently binds to response elements (DR3, DR4) comprising of repeat sequences like AGGTCA that are spaced by three or four nucleotides. It then activates the expression of certain vitamin D target genes.

Synthesis of vitamin D and gene expression is affected by vitamin D receptors that bind to DNA proteins. In the body calcitriol receptors, are responsible for the absorption, production, and regulation of vitamin D, and any alteration in the receptors, increases the risk for different kinds of cancers and other diseases (see Table 2). Patients with CLD exhibit the presence of VDR widely in the liver and inflamed cells. This expression is negatively correlated with disease severity in NASH and CHC patients suggesting a pivotal role of the vitamin D-VDR system in liver damage [71]. During vitamin D synthesis from cholesterol, alterations occur in the DNA sequence, coding for the VDR receptor. It is hypothesized that these calcitriol receptors activate either transcription or gene formation. The RXR is homologous and helps in DNA binding. A network consisting of VDR and the RXR seems to exist. Also the vitamin D system may involve several other receptors and ligands (see Table 2).

The role of vitamin D in the control of proliferation as well as differentiation is reiterated by the target cell levels of 1-alpha hydroxylase. The genomic receptors of concern are targeted by vitamin D to control carcinogenesis [72]. Homeostasis of mineral ions such as calcium, and phosphate is achieved through VDR, and its exact potential in liver, needs to be assessed before promoting it as a therapy [73]. There is significant linkage between VDR polymorphism and incidence of HCC among liver cirrhosis patients. Interestingly SNPs in genes such as FokI, BsmI, ApaI and TaqI genes of VDR were observed [74]. An absence of protective allele A-T-C of Bat gene was found to be linked with HCC. Also a strong linkage between the A-T-C and G-T-T halotype of BAt is observed in HCC [31, 74]. In another study conducted in the Chinese population, it was observed that polymorphism in VDR and DBP gene increases the sensitivity of HBV related HCC. Particularly, VDR rs2228570 and DBP rs7041 polymorphism are more responsible for higher susceptibility to HBV related HCC [22].

The critical molecular signaling pathways attributed to tumor development and its progression in HCC include the MAPK, PI3K/AKT/mTOR, WNT/β-catenin and IGF pathways, and the growth factor-regulated angiogenic signaling. Unfortunately, there is a lack of approved drug so far, which effectively targets the very crucial WNT/β-catenin pathway in HCC. Though sorafenib (nexavar) is currently the sole effective systemic remedy available for HCC, much information is yet wanting [75].

Cyclin proteins are known to be responsible for the proliferation of cells. The expression of p21 and p27, inhibitors of the cyclin-dependent kinase are upregulated in the presence of vitamin D with a concomitant downregulation of IGF1 signaling which reduces mitogenesis. This is in turn achieved by the increased expression of IGF-binding protein 3, transforming growth factor-β or epidermal growth factor. It has been shown that proto-oncogenes such as MYC and KCNH1 are repressed by vitamin D thereby making it an important player in combination therapies against cancer. Vitamin D and its analogs have also been shown to reduce the activity of the enzyme telomerase reverse transcriptase which is overexpressed in cancers. It has been noted that vitamin D induced mRNA miR-498, downregulates the enzyme telomerase reverse transcriptase and promotes cell death thereby reducing the progression of tumors [76].

The phenotype of some malignant cells is altered by vitamin D which in turn promotes cell maturation and cell differentiation. The vitamin has been known to regulate metastasis and enhance the expression of E- cadherin, a tumor suppressor gene [77].

## Vitamin D analogs

Though epidemiological evidence is still inconclusive, biochemical research points out towards a positive association between the inhibitory effects of vitamin D and its analogs in controlling HCC [11, 78]. One such analog EB1089 was found to be inhibitory in controlling the proliferation, and inducing apoptosis of HepG2 cells through VDR causing an elevated expression of p27Kipl and PTEN gene protein levels [79]. In vitro studies show that EB1089 exhibits effective and proliferative properties in malignant as well as pre malignant cells without the side effects of vitamin1,25-(OH)(2) D(3), making it a potential candidate for chemoprevention. EB1089 inhibitory activity is associated with alteration of cell cycle checkpoints through Skp2-dependent p27 induction in Hep-G2 cells [80] Mice (C3H/Sy) treated with EB1089 for chemoprevention showed statistically significant inhibition on HCC incidence [81]. Inactivation of the concerned genes at the preliminary stage could help in apoptosis and further development of malignancy as HCC is quite resistant to chemo preventive interventions [82]. Vitamin D analogue Seocalcitol (EB 1089) have exerted significant progress in Phase 2 clinical trials for the effective treatment of HCC particularly in early stage [83]. Compared to the active form of vitamin D, EB1089 is 50–200 times more potent in vitro as well as in vivo and also show reduced calcaemic activity in vivo [84]. Another Vitamin D analogue, 19 Nor -2α (MART-10) has shown potential cell regulating property with increased chemotherapeutic potential in liver cancer [13]. MART 10 is equally effective in thyroid cancer cells, head and neck cancers, and squamous carcinoma as well [11, 85].

The uptake of vitamin1, 25-(OH) 2D3 by liver tumors, and its retention was found to result in significant reduction of human HCC xenografts without hypercalcemia in nude mice [86, 87]. Clinical results by [88] have also shown the anti-proliferative action of VDR. Though the mechanisms have not been clearly understood, in vitro studies suggest a possible mechanism for VDR-cancer growth inhibition by stimulating apoptosis [89]. In a remarkable study it has been found that 1, 25 D3 protected osteoblasts from theophylline induced apoptosis and also reduces oxidative stress [90].

## Factors contributing towards vitamin D deficiency, and its supplementation

The current recommended intakes of vitamin D appear to be much lower than actually required by the body according to many researchers. A 1000 IU vitamin D would be ideally required to increase blood vitamin D levels to 30 ng/ml considering its absorption, conversion to the active form, and its assimilation by the body [41, 91, 92]. It depends on baseline 25OHD concentration. Supplementation of 1000 IU/d causes a mean increment of 11 ng when the baseline is 10 ng/ml, around 8 ng/ml when baseline level is 30 ng/ml, 5 ng/ml for baseline 50 ng/ml and 1.6 ng/ml for baseline 90 ng/ml. Above a starting value of 90 ng/ml, the response is nearly flat at about 1.6 ng/ml/1000 IU/d [93].

The increased response in serum vitamin D levels upon increasing vitamin D supplementation is not linear for unidentified reasons. It depends upon the baseline serum levels of the vitamin. Increasing the serum vitamin D value to > 50 nmol/L necessitates more requirement of the vitamin than increasing it from a baseline level of < 50 nmol/L. Also the rise in serum vitamin D is steeper on administration of a dose of < 1000 IU/d, and lesser response on administration of higher doses every day. On administration of a dose of < 1000 IU/d, a rise of 1 nmol/L for every intake of 40 IU is observed. On administration of a dose of ≤ 600 IU/d, serum 25(OH) D levels rose by about 2.3 nmol/L for every intake of 40 IU [94].

Many factors like exposure to sunlight at different times of the day, seasons and latitudes, seem to influence the assimilation of vitamin D. Also the type of clothing worn [95, 96] use of topical creams acting as sunscreens, degree of skin pigmentation, age, obesity, and other related chronic illnesses appear to play a significant role in the bioavailability of vitamin D [95]. When the influence on vitamin D by different colors and fabrics was checked for, it was observed that UVB irradiance decreased by 98.6% with black wool as compared to 47.7% with white cotton. However synthesis of vitamin D3 was totally suppressed in vitro by both fabrics upon 40 min of exposure to simulated sunlight.

Although low levels of vitamin D are observed in most of the population in Middle East women in particular are more vulnerable due to their dressing style and tend to exhibit lower levels of the vitamin D as compared to men. The lack of exposure to sunlight by females, coupled with the prevalent cultural practices, may constitute one of the key risk factors to hypo-vitaminosis [97].

Concentrations of 25(OH) D due to supplementation of vitamin D has been found to be inversely associated with increases in body fat percent and BMI [98]. The suggested mechanism is that as vitamin D is a fat soluble vitamin, deposited and stored in the body fat tissue. Therefore larger volumes of adipose tissue tend to trap more amounts of 25(OH) D circulating in the body [99]. People with fat malabsorption, or on extremely low fat diets experience vitamin D deficiency as this vitamin is fat soluble [100]. Excess fatty deposits in the obese also reduce the affectivity of vitamin D absorption. People having undergone gastric bypass surgery are devoid of a portion of the small intestine leading to vitamin D malabsorption [101], People consuming steroids [102], weight-loss drugs [103] and epileptic drugs should be cautious as they could reduce calcium absorption which could significantly impair vitamin D metabolism.

Calcipotriol, a synthetic form of vitamin D inhibits fibrosis in mouse liver cells [104]. The synthetic vitamin D analog could prove better, as it is less labile as compared to the natural vitamin D which is also available in minor quantities in food products. Also the chances of hypercalcemia due to large consumptions of natural vitamin D are decreased as calcipotriol evokes strong responses even in the absence of blood calcium [104].

Many observational studies have correlated the ultraviolet B rays of the sun to be beneficial in reducing the risk of different cancers. Liver cancers were found to be inversely correlated with latitude [105]. In a recent study, sources like sunlight observations, US cancer statistics, Wide-ranging Online Data for Epidemiologic Research (WONDER) data, and CDC data were used to interpret the association between solar energy and many cancers. Solar energy was more strongly linked to cancer occurrence than to mortality. Nevertheless a study of different races and their ethnicity would help us garner more information from this kind of ecological study. The likely reason for the difference could be multifaceted leading to the impairment of the immune system and viral infection, thereby increasing the risk of these cancers [106]. The national cancer institute NCI maps displaying atlas of mortality due to all cancers from 1970 to 2004 in the white, non-white, black, and other combined population irrespective of gender, and adjusted with age suggest that cancer mortality is increased amongst people residing in states along the southern US near the Mexico border. Cancer mortality was also observed in the other southern states of US, which are more populous regions (https://ratecalc.cancer.gov/ratecalc/). Higher rates of liver cancer with increased NOS too is observed in the population residing in this region. This could be due to a high latitudinal gradient with NOS increases. Also prevalence of hepatitis is observed among the Mexicans and Hispanics living along the US Mexico border. Nevertheless cancer effects due to solar radiation mainly being long term increase the complexity of the issue. Moreover cancer related information is not without potential errors, and limitations, and the association between solar radiation and cancers is influenced by environmental as well as genetic factors.

As ample evidence shows that very low doses of sunlight exposure are beneficial without any noted dysfunctions of tissue, more comprehensive photo-immunological studies with various environmental interactions need to be done [107].

It is contemplated that the benefits and dangers due to UV exposure may be argumentative due to skin cancer debates. Still sun avoidance could well compromise sufficiency of vitamin D [108]. Oral vitamin D supplementation could therefore well be a quantifiable good source than UV exposure [109] .

Fish oils like cod liver oil, fatty fish like herring, mackerel, sardine, tuna, and oysters are good sources of vitamin D.Beef liver, cheese, egg yolks, and certain mushrooms also contain vitamin D though to a small extent. Foods like milk, orange juice, soy products, and cereals are fortified with vitamin D, but caution should be exercised in not consuming excess vitamin D intakes to avoid toxic adverse effects. Certain foods like whole milk, cheese, caviar, oysters, salami, and eggs containing vitamin D also contain large amounts of cholesterol and should be consumed in moderate amounts and should be avoided by people at risk of cardiovascular problems.

Inconsistent reports of 25(OH)D measurements by different laboratories, has led to the formation of the Vitamin D Standardization Program (VDSP) in 2010, to establish new standard guidelines for clinical and public health usage of vitamin D [110]. A 4000 IU/d supplementation of the vitamin is considered safe for pregnant women and quite effective in attaining sufficiency in them as well as their neonates irrespective of racial differences [111]**.** It was also observed that a hike in vitamin D levels towards 40 ng/mL in pregnant women reduced the of preterm birth risk and also attain maximal production of the vitamin in its active form [112]. A total intake through all sources has been suggested as 7000 IU/day which is well below any observed adverse effect level of the IOM as well as the Endocrine Society and the recommended safety range by Hathcock et al. [113].

Also certain recommendations have been laid by the Dietary Guidelines for Americans in 2005. People with certain conditions need to be supplemented with vitamin D due to their inability to produce sufficient quantities of the active form of the vitamin. Some food items and supplements also possess the potential to reduce vitamin D deficiency. Glycine has potential to provide protection against bile duct ligation induced liver injury by decreasing oxidative stress, apoptosis and vitamin D deficiency [114, 115]**.** Breastfed infants should get at least10–20 min exposure to the sun to ensure sufficient intake of vitamin D as breast milk is devoid of this vitamin. [116].

A RCT was conducted by Hollis et al. wherein post-partum supplementation of different doses (400 IU, 2400 IU, and 6400 IU) of vitamin D3 to lactating women was done for a period of four to 6 weeks. Only the infants belonging to the 400 IU group received an additional vitamin D supplementation of 400 IU vitamin D3/d.It was observed that a supplementation of 6400 IU/day of the vitamin D safely provides an adequate quantity of the vitamin to breast milk satisfying the baby’s need [117].

Skin aging in the elderly decreases its ability to convert vitamin D from the sun to its active form. Also the pigment melanin usually present in larger quantities in dark skinned people lowers the formation of vitamin D by the body, thereby making fairer people require lesser vitamin D as compared to their darker counterparts [99]**.** Effects of intakes of dietary calcium have been studied with mixed results. The few studies showing increases attribute this either to decreases in the concentration of 25-hydroxylase by 1, 25(OH) D due to a negative feedback process, or lowered intake of the substrate and a slow removal of the vitamin 25 (OH) D by the liver**.** Aging has been also associated with low amounts of vitamin D and lowered absorption of the vitamin through the epidermis. Surprisingly a comparison between young and aged population on supplementation of vitamin D, elicited a significant increase in circulating vitamin D levels in both groups [99]. Also supplementation with estrogenic contraceptives may boost hepatic hydroxylation of this vitamin thereby elevating the circulating VDBP concentrations [99]**.**

More people suffer from insufficiency of vitamin D than excessive intakes. Nevertheless excessive intakes from either food or supplements could lead to calcification of the kidneys, heart and lungs due to high calcium levels in the blood. The resultant calcification could cause arrhythmia, kidney stones, polyuria, fever, anorexia, confusion and pain to the individuals [118]. Usually 800 to 1000 units of vitamin D per day is prescribed as majorly people are deficient in this vitamin. The Institute of Medicine- IOM (now-Health and Medicine Division HMD) recommends an intake of 600 IU/day for 1–50 year olds. Reconsideration of the dosages is awaited based on outcome researches that are in progress [119].

Though hypervitaminosis D is rare, two trials have shown toxicity at 40–60,000 units per month, causing symptoms of polyuria, kidney stones, anorexia and pain in individuals. A supplementation of large quantities of the vitamin has also been found to upset the immune responses [120]. Regular supplementation of a dose as high as 2000 IU of vitamin D per day to infants led to an increased prevalence of allergic rhinitis, atopy and asthma at a later age of 31 yrs. In another study, a supplementation of 400 IU/d vitamin D along with vitamin A (1000 IU) in the first year of infancy, increased the susceptibility to food hypersensitivities and asthma by almost twofold.Certain people with CVD might experience, an adverse effect with higher concentrations of 25(OH)D. This may be also observed in people who have been having an insufficient vitamin D status for years and later in life took supplements as a means of correction [121].

It is possible that genetically some people are more predisposed to having higher levels of vitamin D. According to Carlberg et al., depending upon the variations and alterations in epigeneticity and genetic transcription of blood immune cells, proteins or serum metabolites, individuals could be classified into low, mid or high responders to the vitamin intake.Thus, the concept of personal vitamin D response index should be considered as a valuable indicator instead of only the vitamin Dstatus [122]. So targeting the same dosages for all individuals may not be the appropriate way. Gut microbes in the gut lining are also known to produce quite a few vitamins and these microbes respond to these vitamin receptors as well, thereby affecting their concentrations in the body. So gut health also would reflect the status and concentrations of the vitamin in the body [123]. Vitamin D fortified foods do not pose as much as a risk of vitamin D toxicity as compared to vitamin D dietary supplements. The threshold for children less than 1 year of age for vitamin D through foods and supplements combined is 25 μg (1000 IU) per day and for people of other ages is 50 μg (2000 IU) per day. These levels mostly do not result in any adverse reactions [124]. Although dietary sources provide the vitamin D, it gets converted to the active form in the body only on exposure to sunlight. Ironically increased sun exposure has also been linked to skin cancer prompting experts to advise usage of sun protectors during moderate to high UV levels. But this is controversial as sun exposure and UV absorption is also dependant on factors like skin color, latitude, and season. A brief daily exposure to UV and vitamin D supplement intake has been suggested by some researchers to ensure adequate vitamin D production, and avoid the risk of skin cancer. It has been noted that as compared to vitamin D in the form of either powder or in ethanol, its concentrations in the blood increased when fish oil was used as a vehicle [125]. Earlier studies were proved unsuccessful in establishing a conclusive association between the dietary fat concentrations and its response to supplementation, though certain types of fat made a difference Being a fat soluble vitamin, it requires fat as a vehicle to be absorbed by the body. It was proposed that certain fatty acids like linoleic and linolenic acid may enhance the solubility of vitamin D in the micelles thereby increasing the size of the micelles. This helps in retaining the vitamin which would have to struggle in passing through the intestinal mucosa [126]. Therefore fortifying foods with vitamin D could well decrease the current gap of nutritional intakes of the vitamin worldwide with far reaching impacts [124].

## Other helpful therapies in alleviating liver disorders

Usually anti-inflammatory, anti-fibrotic or immunomodulatory drugs dominate the forefront of liver injury treatment. Drug interactions and toxicities have encouraged modified nutritional uptake, in conjunction for safe and effective treatments. NAFLD displays a malabsorption of fat leading to abnormal bile acids [127]. Failure of the hepatic system leads to impairment of nutrient metabolism. Therapies with omega-3 fatty acids, vitamin E, and vitamin D showed positive effects in NAFLD [128, 129]. The precursor omega-3 fatty acid, ALA, is responsible for synthesizing the potent forms DHA & EPA in the liver. Therefore, a severe deficient status of omega-3 fatty acids is observed in patients with alcoholic cirrhosis [130]. Supplementation with cod liver oil containing both omega-3 fatty acids as well as vitamin D may possibly have a combined synergistic effect in exhibiting anti-cancerous effects through anti-inflammation, anti-proliferation, pro-apoptosis, and anti-angiogenesis effects [131].

## Use of nutraceuticals

Consuming health supplements, and nutraceuticals is the trend of the health seeking consumer. Nutraceuticals have emerged from health enhancing supplements to those preventing disease. Different phytochemicals have been found to be of great potential use in the nutraceutical’s industry as they are not only pharmacologically safe, but they also provide benefits in alleviating the adverse effects of chemotherapy and radiotherapy.But insufficient data makes it difficult to either refute or support the data of supplementation of nutraceuticals in the alleviation of liver disorders [132].

Though tocotrienols have been shown to normalize the echogenic responses in hepatic cells, the LFT’s and lipid profile remained unchanged after supplementation when compared with a placebo [133]. A combination of vitamins C&E with atorvastatin for 4 years was found efficient in reducing hepatic steatosis in NAFLD patients. But the benefits could not be attributed to any single compound. Polyphenols on the other hand appear to exhibit hepato-protective effects by reducing oxidative stress, inflammation and insulin resistance thereby decreasing progression of NAFLD to NASH.

Silymarin from the plant *Silybum marianum* has been proposed as a complementary treatment due to its membrane stabilizing as well as antioxidant potential. Supplementation with resvetrol showed conflicting results with improvements in a 3 months supplementation period as when compared to an 8 weeks period. Anthocyanins, and carnitine too have shown antioxidant potential but further studies are warranted to establish dosages, safety and efficacy [132].

Adverse effects of chemotherapy& radiotherapy have been known to be reduced with the use of herbs like *Ginkgo biloba* and *Emblica officinalisis*, possibly due to their potential antioxidant capacity. Genetic mutations have been arrested and apoptosis induced in cancers such as HCC to halt the progression of the disease [134].

Improved efficacy has been observed with combination therapies such as polyphenols, carotenoids, and flavonoids. It also consists of pigments which influence the redox-regulated and inflammatory pathways without any known toxic effects. Also all types of teas whether black, green tea, or chamomile tea have been shown to modulate the action of hepatic cytochrome P450 sub CYP1A2 [135].

## Role of fiber, vitamins and minerals

Soluble vegetable fiber sources like legumes are known to aid in the fixation of nitrogen through bacteria and decrease the formation of ammonia. Thereby protein tolerance is increased resulting in enhanced immunity that is required in liver disorders [136] .

Zinc is responsible for the production of urea in the liver [137]. Deficiencies of zinc, water soluble vitamins, s-adenosyl methionine (SAMe) are also observed in CLD [138]. SAMe triggers the immune system, and decreases hepatotoxicity due to TNF alpha production [139]. Also, decreased concentrations of SAMe correlates with decreased mitochondrial concentrations of the potent antioxidant glutathione [140]. Paracetamol in overdoses has shown to produce severe liver cell injury causing depletion of glutathione, protein alkylation, and an increase in reactive oxygen/nitrogen species leading to hepatocellular necrosis [141]. The fat soluble vitamin E along with vitamin D when supplemented orally or parenterally is effective in improving the status of deficiency in chronic conditions of cholestasis [142]. Though many studies point towards a positive response to vitamin D and mineral supplementation, insufficient evidence exists to prove its benefits to encourage their usage in prevention of liver cancer and CLD [143].

## Anabolic steroids and growth hormones

Use of steroids was found effective only in moderate cases. But in severe malnourishment no beneficial effects were observed [144]. Also interventions with growth hormones was found to only increase IGF1 & IGFBP-3 levels in patients with liver cirrhosis and parameters related to BMI leaving those of liver function unaltered [145]. It is concluded that deficiency of vitamin D may affect IGF-1 level in children and adolescents exhibiting deficiency of growth hormones like IGF-1 [146]. VDR polymorphism has also been associated with idiopathic short stature in several studies [23].

Recently Marek et al. have conducted an important study linking growth hormone (IGF-1 axis), vitamin D and bone mineral density (BMD) in patients afflicted with chronic viral hepatitis. They observed a positive correlation between bone mineral density & IGF-1, and IGFBP-3 & BGLAP levels. Chronic viral hepatitis led to a reduction of IGF-1 and IGFBP-3 and increase in GH secretion [24]. In a remarkable study it has been found that 1, 25 D3 protected osteoblast from theophylline not only by inducing apoptosis, but also by reducing oxidative stress [90]. Hepatic osteodystrophy in children consists of vitamin D deficiency rickets, poor bone mineral density and fractures caused by nutritional imbalances which must be treated with cholecalciferol (D2) or ergocalciferol (D3) [27].

## Role of omega-3 fatty acids

Omega-3 fatty acids EPA, and DHA are known for exerting beneficial effects on metabolic and inflammatory functions and thus may confer specific advantages in liver disorders too. There are recommendations for usage of a combination of 1 g/d fish oil containing omega-3 fatty acids, along with olive oil containing omega-9 fatty acids, 1000 IU/day of vitamin D and 400–800 IU/day of vitamin E in NAFLD treatment [147]. This calls for further researches combination therapies using omega-3 fatty acids and vitamin D in the treatment of liver disorders.

## Role of amino-acids

Increases in the concentration of aromatic amino-acids in cirrhosis cause an imbalance in the ratio of branched and aromatic amino-acids. Tryptophan and the branched amino-acids compete for the same amino acid transporter in the blood brain barrier thereby altering ammonia concentrations in the brain [148, 149]. Experimental studies with glutamine have proven quite beneficial making it a potential candidate for future therapeutics [150]. Some food items and supplements have also the ability to reduce vitamin D deficiency. As mentioned before, glycine has the potential to provide beneficial effects against bile duct ligation induced liver injury by reducing oxidative stress, apoptosis and vitamin D deficiency [114].

## Enteral and parenteral nutrition

As malnutrition is very much prevalent in patients with CLD, the nutritional status needs to be carefully assessed to avoid any other related complications like allograft dysfunction during transplantation [151]. Liver transplantation requires a strong nutritional support during all phases. Enteral as well as parenteral nutrition is recommended to patients with liver ailments like cirrhosis, hepatitis, liver failure or surgical procedures including liver transplantation [152]. The nutritional status is an important factor for survival after liver transplantation due to complications and different medications given [153, 154]. Inflammation and dysfunction in liver diseases have shown better prognosis with proper nutritional intakes and vice-versa [155, 156]. A balance of protein and energy appear to considerably alleviate the existing complications in HCC patients advised chemo-embolization [157]. A Japanese study has positively correlated a better quality of life, low hospital costs and stay, when appropriate post-operative nutritional care was administered [158, 159]. Also protein of vegetable sources was preferred over the animal origin protein in hepatic encephalopathy patients due to controversial reports [160].

## The microbiota connection

A growing body of evidence supports a relationship between gut microbiota and liver diseases like liver encephalopathy, NAFLD, and NASH. A continuous bidirectional communication between the gut and liver exists through the bile, inflammatory mediators, different hormones and products of digestion. This affects the qualitative and quantitative composition of intestinal microbiota on the function of liver and physiology. Therefore manipulations of the gut flora using pre and probiotics could well be considered. Experimental treatments have brought about substantial improvements in the plasma levels of products of oxidative stress namely 4-hydroxynonenal and malondialdehyde in patients with NAFLD and alcoholic cirrhosis. Also decreases in pro-inflammatory cytokine concentrations have been observed in patients with alcoholic liver cirrhosis [161].

Dysfunction in the mitochondrial activity may induce an increased generation of free radicals triggering the production of lipid peroxides and cell apoptosis. An impaired mitochondrial functioning is thought to contribute to NAFLD. Therefore the role of antioxidants targeting the mitochondria has great therapeutic potential.

NAFLD being the one of the commonest of CLD’s globally which may lead to cirrhosis, liver cancers and death, needs proper clinical management. First-line modern clinical management of NAFLD treatment focuses on dietary and lifestyle modifications due to the co-morbidities that are associated with CVD and obesity. Moderate reductions in weight either through proper nutritional management accompanied by regular physical activity is considered safe and also highly recommended. Interventions with antioxidant vitamin E, lipid lowering drugs, insulin sensitizer agents, and intakes of vitamin D3 have also been targeted for therapeutic applications [162].

## Conclusion and future direction

Migration of earlier human race from the African region to the Northern territory made them experience different environmental changes in seasons and sunshine intensities. Therefore, skin pigmentation could possibly be a fine example of natural selection affecting the absorption of vitamin D. The studies done so far reflect deficiency of vitamin D as a marker of health problems. Inflammation during disease could possibly be responsible for lowered amounts of vitamin D thereby explaining its lowered values in a range of disorders. Moreover vitamin D receptors exist in all tissues of the body regulating the immune function and cell proliferation. Therefore the possibility of harm due to intakes gets underscored. It has been noted that numbers of people affected by hyper-vitaminosis accompanied by hyper calcification are the ones who are direct users of Over-the-counter OTC drugs, and not supervised or advised medically, and were consuming more than the Institute of Medicine’s recommendation of a maximum of 10,000 IU/day.Vitamin D has been shown to have therapeutic potential against CLD’s like CHC, liver fibrosis and hepatic necro-inflammation [163]. Vitamin D plays a significant role in the development of NAFLD and containing these related problems. .

As our genetic evolutionary makeup as well as environmental factors seem to influence the levels of vitamin D, we could also put to use this information to understand if naturally low levels of vitamin D actually increases the risk of a particular disease or a consequence of it. Therefore better designed, researches are required to discern specific effects of vitamin D in the reduction of risk of liver disorders and progression to liver cancers and their therapy.

As vitamin D deficiency has taken pandemic proportions, it is essential that healthcare professionals bring about awareness in the population. Sensible guidelines to sun exposure and supplementation of vitamin D are required. Subtle corrections could lead to huge impacts on quality of life and healthcare costs globally [91]. Food fortifications too could be used to increase consumptions of the vitamin and alleviate many a problems faced due to its deficiency [124].

Although a lot is known about the dynamism of vitamin D, the present knowledge needs to be integrated comprehensively and cohesively so as to suggest innovative interpretations of the existing results in the treatment of cancers and liver diseases apart from conducting further researches [1].

## Abbreviations

ALD: Alcoholic liver disease
CLD: Chronic liver disease
DPT: Dermatopontin
FXR: Farnesoid X receptor
HCC: Hepatocellular carcinoma
NAFLD: Non-alcoholic fatty liver disease
NASH: Non-alcoholic steatohepatitis
SAMe: S-adenosyl methionine
VDBP: Vitamin D binding protein
VDR: Vitamin D receptor
