# Correction to: Current therapies in alleviating liver disorders and cancers with a special focus on the potential of vitamin D

**DOI:** 10.1186/s12986-018-0257-z

**Published:** 2018-03-16

**Authors:** Shahida Khan, Ashraf Ali, Sarah Khan, Ahmed Bakillah, Ghazi Damanhouri, Aziz Khan, Ahmad Makki, Ibtehal AlAnsari, Naheed Banu

**Affiliations:** 1 Applied Nutrition and Natural Products Group, King Fahd Medical Research Center, King Abdulaziz University, P.O.Box 80216, Jeddah, 21589 Kingdom of Saudi Arabia; 2 National Brain Research Center, Manesar, Gurgaon, 122051 India; 3 Department of Medicine, SUNY Downstate Medical Center, 450 Clarkson Avenue, Brooklyn, NY 11203 USA; 4 Department of Biochemistry, College of Medical Rehabilitation, Buraydah, Qassim University, P.O. Box 51452, Qassim, Kingdom of Saudi Arabia

## Correction

Following publication of the original article [1] one of the authors wrote to say that his name had been spelt incorrectly as Ahmed Makki instead of Ahmad Makki.

The original article has been corrected.
